# Standing and supine positions are better than sitting in improving rightward deviation in right-hemispheric stroke patients with unilateral spatial neglect: A randomized trial

**DOI:** 10.1097/MD.0000000000031571

**Published:** 2022-11-18

**Authors:** Hitoshi Onaka, Ken Kouda, Yukihide Nishimura, Hidenori Tojo, Yasunori Umemoto, Toshikazu Kubo, Fumihiro Tajima, Yukio Mikami

**Affiliations:** a Department of Rehabilitation Medicine, Wakayama Medical University, Wakayama city, Wakayama, Japan; b Rehabilitation Medicine, Iwate Medical University, Iwate, Japan; c Department of Rehabilitation Medicine, Akitsu Kounoike Hospital, Gose city, Nara, Japan; d Kyoto Prefectural University of Medicine, Kyoto city, Kyoto, Japan.

## Abstract

How body position affects unilateral spatial neglect (USN) is unclear. This cluster randomized trial aimed to examine the effects of different positions (supine, sitting, and standing) on USN in stroke patients.

**Methods:**

Twenty stroke patients (hemorrhage [n = 11], infarction [n = 9]) who were right-handed, had left hemiplegia due to right hemisphere damage that occurred within the last 2 years, and were in a state of arousal with a Glasgow Coma Scale score of 15 were included in the study. Table-top pen-and-pencil tests for USN (Bells Test, Line Bisection, Scene Copy, and Star Cancellation) were randomly conducted in the supine, sitting, and standing positions.

**Results:**

The mean values in each test were significantly smaller in the supine position than were those in the sitting position (*P* = .015, .047, .015, and <.001), and those in the standing position were significantly smaller than those in the sitting position (*P* = .007, <.001, =.006, and < .001). The results of the 4 tests in the standing position were similar to those in the supine position.

**Conclusions:**

Body position affects USN in stroke patients and that the standing and supine positions improve USN better than the sitting position. Some possible mechanisms are: muscle contractions in the lower limbs and the trunk could have affected results in the standing position, and reduction in gravitational stimulation in the supine position could have played a role.

## 1. Introduction

Unilateral spatial neglect (USN) is a phenomenon in which patients with unilateral cerebral disorder or disease tend to neglect visual stimuli presented on the contralateral side of the impaired cerebrum.^[1–3]^ Brain first systematically studied this phenomenon and concluded that as visual neglect is generally seen after right hemisphere damage, it occurs as left-side visual neglect.^[4]^ Neglect is an upper neuron disease characterized by complicated syndromes of perception, motor, and expressive disorders.^[5]^ Most patients with USN cannot perceive or respond to stimuli in the contralateral visual space,^[6]^ show impaired expression of the unilateral space,^[7,8]^ and have decreased use of the contralateral upper and lower limbs.^[9]^ Therefore, USN becomes a serious inhibitory factor in stroke rehabilitation therapy. Rehabilitation therapy for USN includes top-down approaches, which prompt patients to voluntarily explore space by a clue such as hearing and vision, and bottom-up approaches, which make patients involuntarily pay attention to the side of neglect by passive stimulation. In recent years, repetitive transcranial magnetic stimulation and transcranial direct current stimulation have been applied, and the effectiveness of table-top pen-and-pencil tests has been shown.

Early ambulation after stroke has been recommended by several worldwide guidelines in recent years. Studies have shown that gait recovery can be expected by conducting very early mobilization within 24 hours of an acute phase of stroke, which is practicable and safe.^[10,11]^ Studies on high-load/long-term stroke rehabilitation therapy from an early phase by technically proficient therapists under proper medical supervision showed that not only is there a difference in the activities of daily living during the hospital stay in the acute phase, but the rate of home discharge at 6 months after onset also improves, showing that body position may have a favorable effect on recovery after stroke.^[12,13]^ Regarding the relationship between upper limb motor impairments and body position, Tojo et al^[14]^ reported a better improvement in the functional performance of the hand and arm on the unaffected side of patients with right hemiplegia in the standing position compared to the sitting position. Several studies show that USN is also affected by body position. Pizzamiglio et al^[15]^ and Saj et al^[16]^ reported a better improvement in rightward deviation in patients with visuospatial neglect in a decubitus position compared to the sitting position. Meanwhile, Karnath et al^[17]^ reported that a comparison of exploratory eye movements in patients with spatial neglect in various standing positions showed no significant difference, and Mennemeier et al^[18]^ reported no difference in the line bisection test results between decubitus and standing positions. Thus, the effects of body position on USN are controversial and unclear. This study aimed to examine the effects of different positions (supine, sitting, and standing), especially standing, on stroke patients with USN using table-top pen-and-pencil testing.

## 2. Methods

### 2.1. Participants

Twenty patients with stroke (hemorrhage [n = 11, age: 69.1 ± 2.9 years, 55–82 years], infarction [n = 9, age: 73.4 ± 4.1 years, 53–87 years]) were enrolled, comprising 7 men (70.3 ± 4.8 years, 55–87 years) and 13 women (71.5 ± 2.9 years, 53–85 years) (Table 1). All participants had developed hemorrhagic or ischemic stroke in the right hemisphere seen in magnetic resonance imaging or computerized tomography images. Based on the clinical laboratory test results and medical examination, a diagnosis was made by physicians at our department. Of the 20 patients, 19 had left hemiplegia, and 15 had a somatosensory disorder. No patient had sensory loss. The selection criteria in this study were as follows: right-handed before the onset of stroke, 20 years old or older, left hemiplegia caused by right hemisphere damage due to stroke, within 2 years after the onset of stroke, and state of arousal on the Glasgow Coma Scale (GCS) of 15. No participants had reduced visual acuity, motor impairment in the right upper limb, or a history of mental disorder, which may have affected the neuropsychological assessment. This study was approved by the Wakayama Medical University Hospital Institutional Review Board (No. 925), and written informed consent was obtained from all the participants. All procedures performed are in accordance with the ethical standards of the institutional and national research committee and with the 1964 Declaration of Helsinki and its later amendments or comparable ethical standards.

**Table 1:** Subject characteristics.

Subjects	Age	Sex	Education (yrs)	Days since stroke	Etiology	Lesion site (right)	Hemiparesis (left)	Sensory disorders (left)
1	59	F	12	23	Ischemia	Frontal, Parietal	Yes	Yes
2	78	M	12	333	Ischemia	Internal capsule, Basal ganglia, Frontal, Parietal, Temporal, Occipital	Yes	Yes
3	78	F	12	19	Hemorrhage	Internal capsule, Thalamus	Yes	Yes
4	85	F	9	55	Ischemia	Parietal, Temporal	Yes	Yes
5	80	F	6	12	Hemorrhage	Basal ganglia	Yes	Yes
6	63	M	16	16	Hemorrhage	Thalamus	Yes	Yes
7	64	F	9	288	Ischemia	Frontal, Parietal	Yes	No
8	67	F	9	30	Hemorrhage	Basal ganglia	Yes	Yes
9	56	M	16	50	Hemorrhage	Thalamus, Basal ganglia	Yes	Yes
10	85	F	13	15	Ischemia	Frontal, Parietal	Yes	Yes
11	65	F	9	11	Hemorrhage	Basal ganglia	Yes	Yes
12	53	F	12	8	Ischemia	Temporal	Yes	No
13	55	M	9	8	Hemorrhage	Basal ganglia	Yes	No
14	71	M	9	6	Ischemia	Frontal, Temporal	Yes	No
15	87	M	12	34	Ischemia	Frontal, Parietal, Temporal, Occipital	Yes	Yes
16	73	F	14	91	Hemorrhage	Parietal, Temporal, Occipital	Yes	Yes
17	79	F	14	10	Ischemia	Frontal, Parietal, Temporal	No	Yes
18	63	F	14	18	Hemorrhage	Basal ganglia	Yes	Yes
19	78	F	9	20	Hemorrhage	Basal ganglia	Yes	Yes
20	82	M	8	8	Hemorrhage	Thalamus	Yes	No
mean	71.1		11.2	52.8				
SE	2.4		0.6	20.3				

### 2.2. Procedure

The participants were individually tested by an examiner in a quiet room. For the supine position, each participant was asked to lie down on a treatment bed, the head was supported with a pillow, and both legs extended to set a position in which a testing sheet was easily viewed. For the sitting position, each participant was seated on a chair with the backrest fixed using a belt. Each participant’s trunk was extended, hip and knee joints bent at as close to right angles as possible, with the feet on the floor. For the standing position, a stand-in therapy table with which each participant could easily and safely stand was used. Each participant’s trunk and lower limbs were fixed to the stand-in therapy table with a belt. In all the tests, the desk height was set to the height of the olecranon, and the table-top was set perpendicular to the trunk. During testing, in the supine position, all the participants placed their right hand on the sheet in front of them, and in the sitting and standing positions, they placed their right upper limb on the table-top. The order of the 3 positions and 4 tests was randomly set for each participant. Each participant completed all tests on the same day.

### 2.3. Tests

We selected tests that participants were able to be completed in 3 postures on the same day. We used the following 3 tests that were used in a preceding study by Saj et al^[16]^: Bells test,^[19]^ Line Bisection test,^[20]^ and Scene Copy test.^[21]^ In addition, we used the Behavioral Inattention Test, the Japanese version of the Star Cancellation. These 4 tests were conducted in the supine, sitting, and standing positions.

#### 2.3.1. (1) Bells test

We presented a sheet on which outlines of various objects including 35 targets (bells) and others (houses, horses, etc) were drawn. The objects were arranged in random order and distributed equally in 7 columns. Of the 7 columns, 3 were placed on the left side of the sheet, 1 was in the middle, and 3 others were on the right side. We asked participants to circle the targets (bells). The task was terminated at a time when the participant stopped the activity. There was no time limit, and the maximum score was 35. Of 15 bells (left), 5 bells (middle), and 15 bells (right), the number of omitted bells was recorded. The cutoff point was when 3 or more bells of the 15 bells on the left were omitted.^[16]^

#### 2.3.2. (2) Line bisection test

On a sheet, 20 lines parallel to its long axis were drawn. Of those, 18 lines were organized into 3 sets of 6 lines each, and each set contained 100 mm, 120 mm, 140 mm, 160 mm, 180 mm, and 200 mm lines. Each participant was instructed to bisect each line at a position as close to the center as possible. We measured the length of the left side of the line, that is, the length from the left end of the line to the position where the participant was marked, with 0.5 mm accuracy. The measurements were converted to standardized scores by using the following formula:

Deviation = (measured length of the left side—true half-length)/true half-length × 100

A deviation of ≥ 11% was considered as the cutoff.^[16]^

#### 2.3.3. (3) Scene copy test

Participants were instructed to copy the figures of a tree, a fence, a house, and another tree. We scored 1 when only the chimney and opposite window were omitted, 2 when half of the opposite tree and the house were omitted, 3 when the opposite tree was completely omitted, and 4 when the figures on the same side as the opposite tree were omitted. A score of ≥1 was considered the cutoff.^[16]^

#### 2.3.4. (4) Star cancellation test

On a sheet, 52 large stars, 10 short words, and 13 characters were randomly arranged; among them, 56 small stars were interspersed. Participants were directed to cross out all the small stars throughout the page. The maximum score was 54 (27 on the left and 27 on the right). The total number of omitted small stars was recorded. The cutoff was when 2 or more small stars on the test sheet were omitted.^[16]^

### 2.4. Statistical analyses

The results from the 4 tests conducted in the 3 positions were analyzed separately (Bells Test; the number of omitted bells on the left side [/15], Line Bisection; percentage of rightward deviation [%], Scene Copy; score [0–4], and Star Cancellation; the number of omitted small stars on the test sheet [/54]). The data are all presented as mean ± standard deviation. An analysis of variance was performed to evaluate the mean values, with the tests (the 4 tests) and positions (standing, supine, sitting) considered as parameters. When the result of an analysis of variance test was significant (*P* < .05), the Tukey test was used as a post hoc comparison. *P* values < .05 were considered significant.

## 3. Results

**Figure 1.**
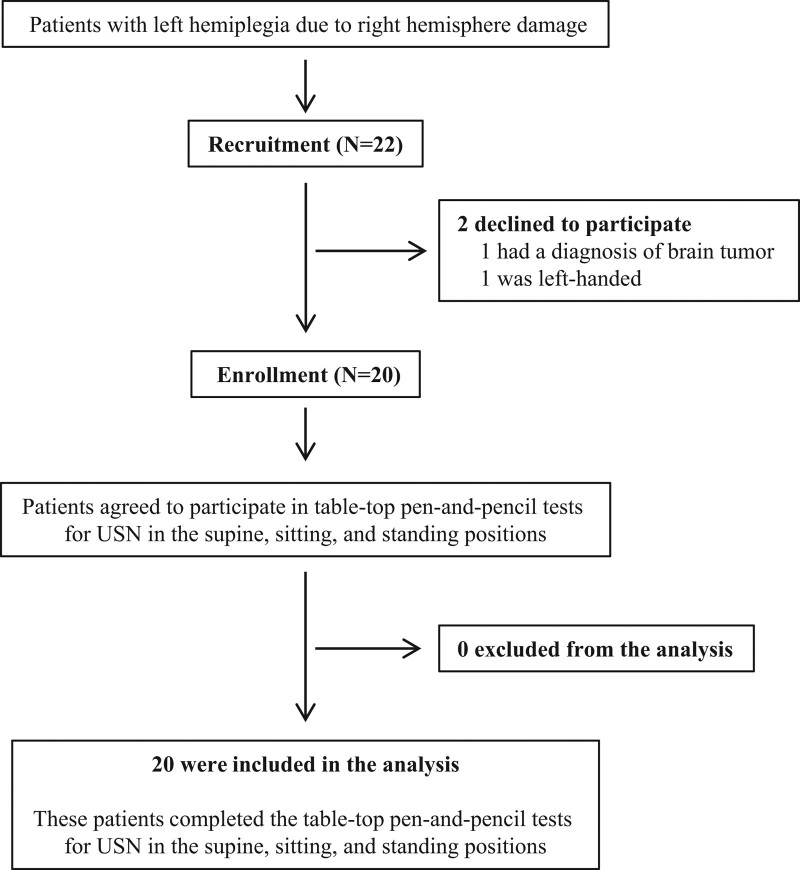
Consort flow diagram for the enrollment of participants in this study.

A consort flow diagram of this study is shown in Figure 1. Twenty-two patients with left hemiplegia due to right hemisphere damage were assessed for eligibility, and finally, 20 patients were enrolled in this study (Fig. 1). Of the 2 patients excluded, one had a brain tumor diagnosis, and the other was left-handed. Every participant could complete all the tests, and there was no deviation in the order of the 3 positions. The results of the 4 tests in the 3 positions are summarized in Table 2. The results of each test varied in the supine, sitting, and standing positions, and the interactions were significant (*P* < .001, 001, 001, and .001). In the previous study by Saj et al,^[16]^ when the test result was a cutoff value in 2 or more tests of the 3 tests (Bells, Line Bisection, and Scene Copy tests), it was judged that the participant had USN. Based on these criteria, all participants in our study were judged to have USN in the sitting position, while 8 participants in the supine position and 10 in the standing position were judged to have USN (Table 2).

**Table 2:** Results of 4 tests for USN conducted in 3 positions.

Subjects	Bells test			Line bisection (%)			Scene copy			Star cancellation		
	SU	SI	ST	SU	SI	ST	SU	SI	ST	SU	SI	ST
1	2	4	4	21.2	29.0	27.6	0	1	0	3	8	4
2	15	15	15	24.3	47.4	40.2	4	4	4	48	43	34
3	14	15	15	30.4	12.9	8.5	4	4	0	21	15	28
4	15	15	15	82.5	72.6	42.9	4	4	4	45	47	42
5	15	15	15	70.6	17.9	21.4	4	4	4	37	38	39
6	14	5	7	-1.2	35.6	3.0	0	0	0	19	6	4
7	3	5	0	15.1	9.9	8.8	0	2	0	9	12	2
8	14	4	3	6.8	12.7	-0.5	1	2	0	42	2	2
9	1	5	0	-2.6	12.0	-6.5	0	0	0	0	0	0
10	2	7	3	5.3	17.5	3.7	0	0	0	2	44	1
11	15	15	15	94.8	74.3	55.3	4	4	4	46	46	41
12	2	6	3	-0.2	14.5	0.7	0	0	0	1	0	0
13	1	10	3	4.3	18.9	17.6	0	0	0	0	14	4
14	2	15	2	0.6	15.2	-2.4	0	0	0	0	2	0
15	11	15	11	68.1	84.2	33.3	3	4	3	42	50	40
16	7	15	8	20.8	32.8	11.5	0	4	0	4	9	5
17	0	5	0	6.2	17.5	1.2	0	1	0	1	10	0
18	3	15	12	12.5	55.0	31.5	0	3	0	8	28	17
19	13	15	15	-17.6	38.6	5.4	0	4	3	16	40	23
20	3	13	0	7.3	40.1	0.3	0	0	0	0	5	0
Mean	7.6	10.7	7.3	22.5	32.9	15.2	1.2	2.1	1.1	17.2	21.0	14.3
SE	1.4	1.1	1.4	7.0	5.1	3.9	0.4	0.4	0.4	4.2	4.1	3.7

SI = sitting, ST = standing, SU = supine, USN = unilateral spatial neglect.

**Figure 2.**
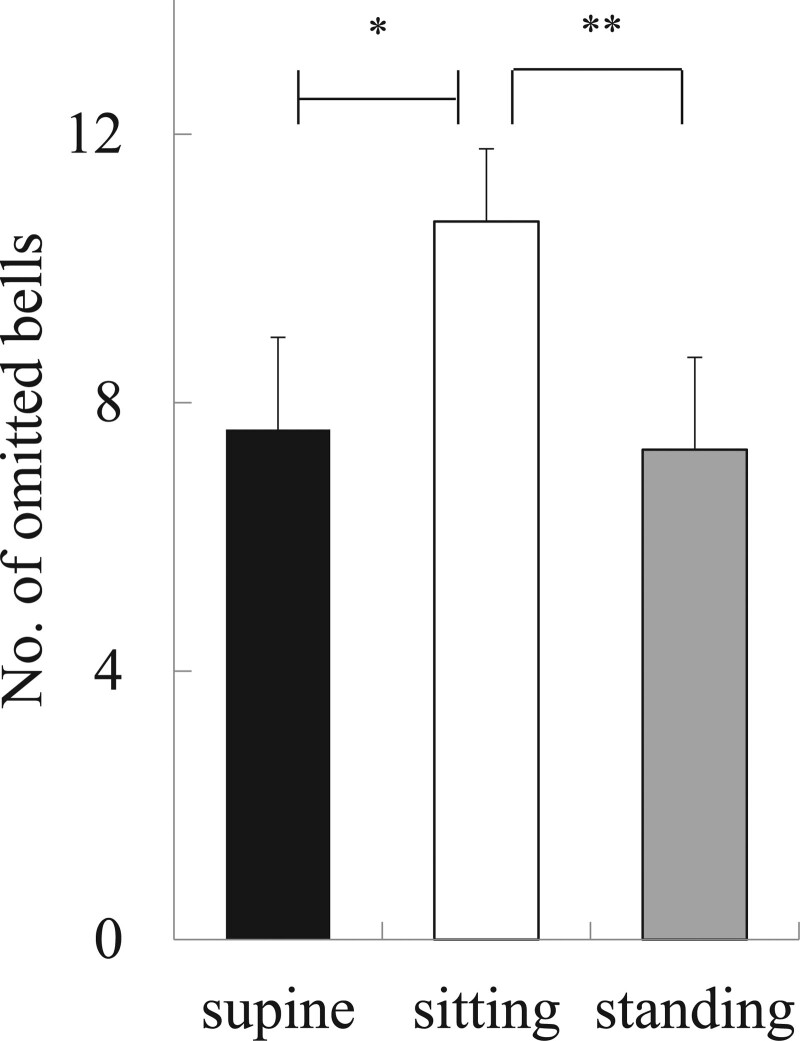
Results of the Bells test. The number of omitted bells was 7.6 ± 1.4 on average in the supine position, which was significantly smaller than 10.7 ± 1.1 on average in the sitting position (^*^P < .05). The mean value in the standing position, 7.3 ± 1.4, was significantly smaller than in the sitting position (^**^P < .01). The results of the Bells test in the supine position were similar to those in the standing position.

In the Bells test, the number of omitted bells was 7.6 ± 1.4 in the supine position, which was significantly smaller than 10.7 ± 1.1 on average in the sitting position (*P* = .015). The mean value in the standing position, 7.3 ± 1.4, was significantly smaller than in the sitting position (*P* = .007). The results of the Bells test in the supine position were similar to those in the standing position (Fig. 2). In 14 participants, the number of omitted bells was smaller in the supine position than in the sitting position, and in 12 participants, the number was smaller in the standing position than in the sitting position (Table 2).

**Figure 3.**
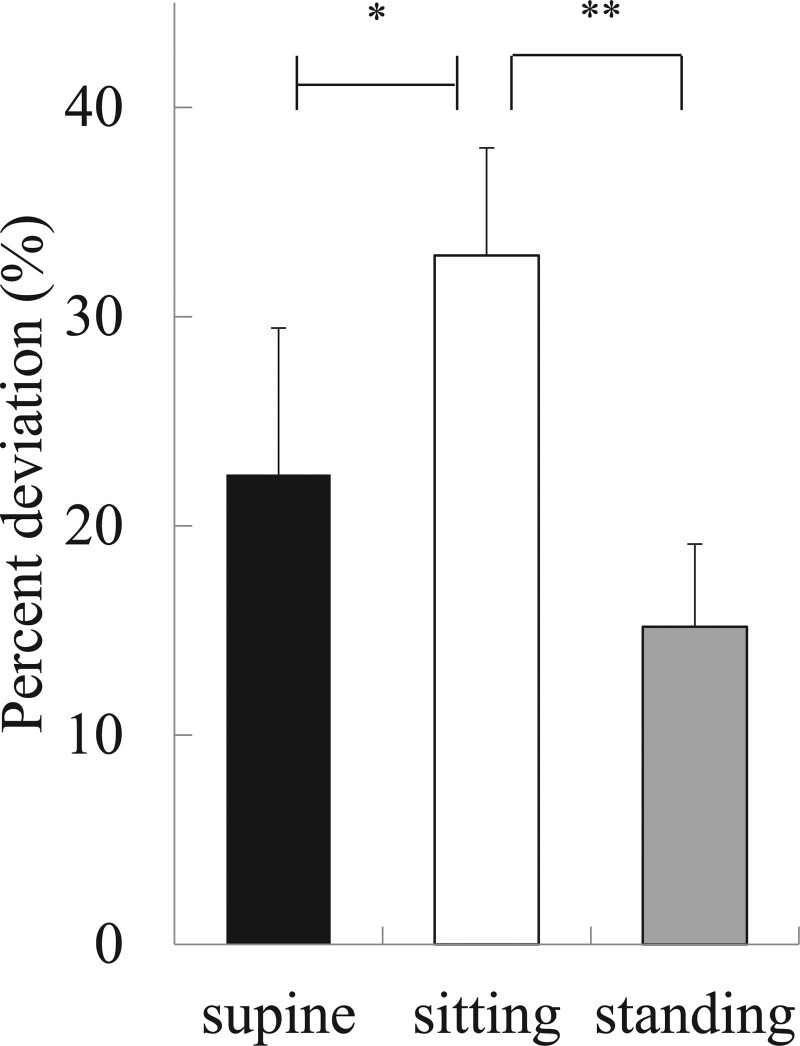
Results of the Line Bisection test. The mean rightward deviation was 22.5% ± 7.0% in the supine position, which was significantly smaller than 32.9% ± 5.1% in the sitting position (^*^P < .05), and the value in the standing position, 15.2% ± 3.9%, was significantly smaller than in the sitting position (^**^P < .001). The results of the Line Bisection in the supine position were similar to those in the standing position.

In the Line Bisection test, the mean rightward deviation was 22.5% ± 7.0% in the supine position, which was significantly smaller than 32.9% ± 5.1% in the sitting position (*P* = .047), and the value in the standing position, 15.2% ± 3.9%, was significantly smaller than in the sitting position (*P* < .001). The results of the Line Bisection test in the supine position were similar to those in the standing position (Fig. 3). In 15 participants, the rightward deviation was smaller in the supine position than in the sitting position, and in 19 participants, it was smaller in the standing position than in the sitting position (Table 2).

**Figure 4.**
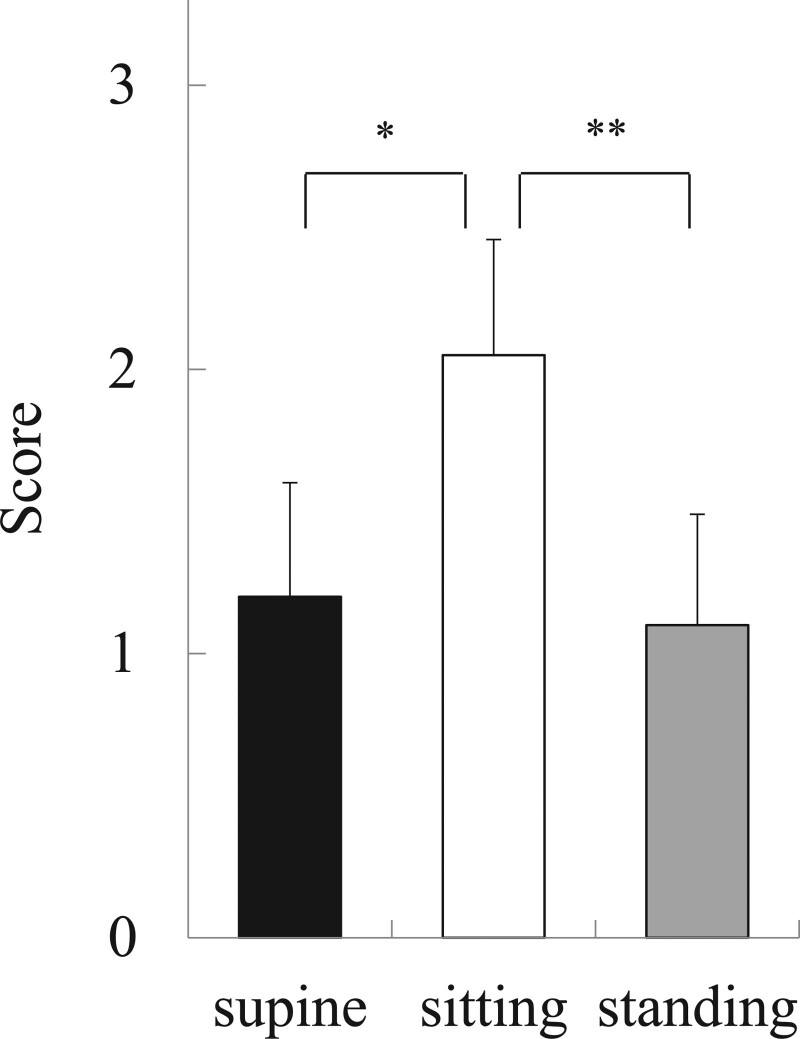
Results of the Scene Copy test. The mean score was 1.2 ± 0.4 in the supine position, which was significantly lower than 2.1 ± 0.4 in the sitting position (^*^P < .05). The score in the standing position, 1.1 ± 0.4, was significantly lower than in the sitting position (^**^P < .01). The results of the Scene Copy test in the supine position were similar to those in the standing position.

In the Scene Copy test, the mean score was 1.2 ± 0.4 in the supine position, which was significantly lower than 2.1 ± 0.4 in the sitting position (*P* = .015), and the score in the standing position, 1.1 ± 0.4, was significantly lower than in the sitting position (*P* = .006). The results of the Scene Copy test in the supine position were similar to those in the standing position (Fig. 4). In 8 participants, the score was lower in the supine position than in the sitting position; in 9 participants, it was lower in the standing position than in the sitting position (Table 2).

**Figure 5.**
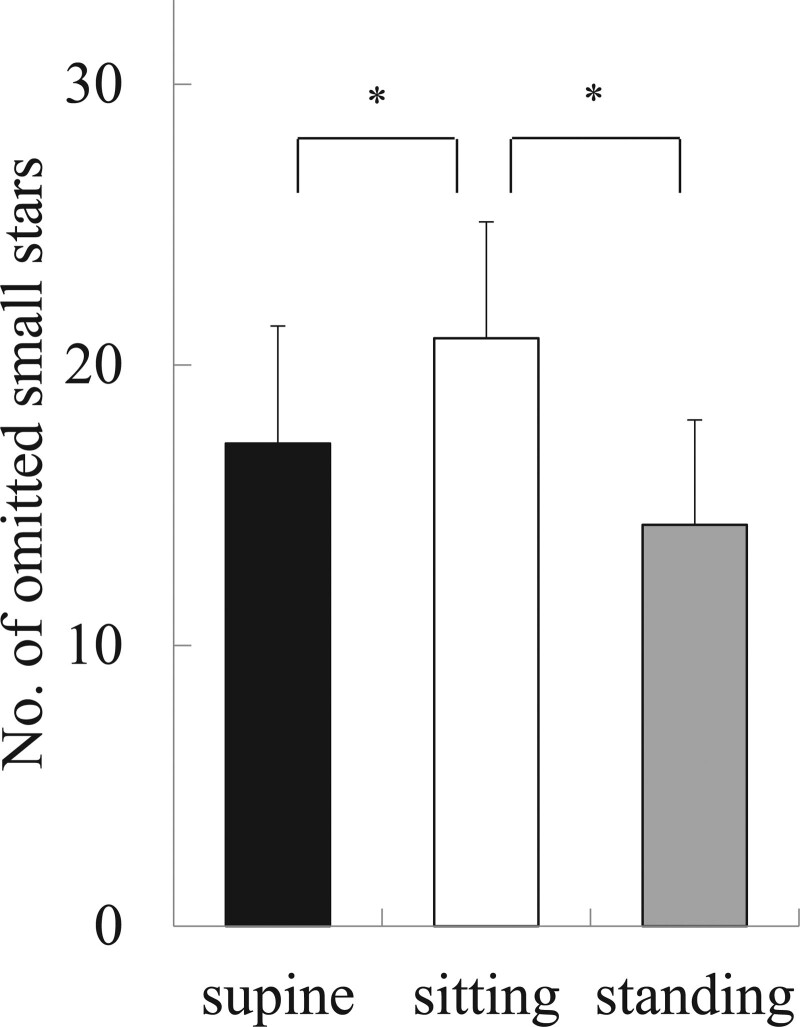
Results of the Star Cancellation test. The number of omitted small stars was 17.2 ± 4.2 in the supine position, which was significantly smaller than 21.0 ± 4.1 in the sitting position (^*^P < .001), and 14.3 ± 3.7 in the standing position, which was significantly smaller than in the sitting position (^*^P < .001). The results of the Star Cancellation test in the supine position were similar to those in the standing position.

In the Star Cancellation test, the number of omitted small stars was 17.2 ± 4.2 in the supine position, which was significantly smaller than 21.0 ± 4.1 in the sitting position (*P* < .001), and 14.3 ± 3.7 in the standing position which was significantly smaller than in the sitting position (*P* < .001). The results of the Star Cancellation test in the supine position were similar to those in the standing position (Fig. 5). In 13 participants, the number of omitted small stars was smaller in the supine position than in the sitting position, and in 15 participants, it was smaller in the standing position than in the sitting position (Table 2).

## 4. Discussion

This study examined the effects of body position on table-top pen-and-pencil tests for USN in right-hemispheric stroke patients. It demonstrated that the standing and supine positions improved rightward deviation better than the sitting position. Therefore, body position was shown to affect USN. In the standing position, contraction of the antigravity muscles, muscles of the lower limbs, and the trunk conceivably affected arousal and attention through the ascending reticular activating system. In the supine position, a reduction in gravitational stimulation may have reduced asymmetric abnormal signal input through the vestibular nervous system.

Most patients with right hemisphere damage in whom unilateral cognitive impairment is accompanied by rightward deviation show immanent arousal impairment.^[22]^ Functional imaging and animal experiments have shown that the cortical region in the right hemisphere governs the control of imminent arousal and continuous attention. Sturm et al^[23]^ have pointed out that both immanent arousal and continuous attention share the network of the frontal lobe and the parietal lobe in the right hemisphere. In this study, as all the participants were visuospatial neglect patients with lesions in the right hemisphere, we only included patients with a high level of arousal (GCS = 15 in all the participants) to prevent varied arousal levels. In addition, to prevent variations caused by the test procedure, we conducted testing on the same participants on the same day and randomly chose participants’ positions and test items. We considered that uniformizing the arousal state in this study’s population and adjusting the test procedure allowed an accurate assessment of the effects of body position on spatial neglect.

A unification of somatic sensations such as cutaneous and deep sensation, visual sensation, and vestibular sensation in the brain play an important role in humans perceiving space. Such sensory information is shown to vary with body position, such as supine, sitting, and standing, affecting the function of spatial perception in USN patients. Pizzamiglio et al^[15]^ reported a reduction in the pathological deviation in USN patients in the supine position rather than in the sitting position. As a mechanism, they state that placing patients with abnormalities in the vestibular sensory system, which percepts left-right symmetry by gravitational stimulation, in the supine position allows a reduction in the input of its abnormal signals to improve USN.^[15,24]^ Saj et al^[16]^ reported that when USN patients were asked to perform a task in the dark to eliminate the influence of visual information, there was a significant rightward deviation in the sitting position, whereas, in the supine position, it largely decreased. They stated that in the supine position, reduction in the input of gravitational stimulation, which supports the trunk, leads to decreasing pathological deviation in USN patients. Thus, gravitational stimulation and the vestibular sensory system affect the processing of spatial information. In contrast, Funk et al^[25]^ reported that rightward deviation in USN patients is worse in the supine position compared to the sitting position and that the strongest rightward deviation is observed in USN patients in whom the state of arousal is particularly low. Funk et al^[25]^ also stated that in the supine position, a reduction in the level of arousal in USN patients might have affected their spatial deviation. In addition, studies in healthy individuals have shown that reduction in the level of arousal causes symptoms such as USN.^[26,27]^ When USN patients wake in stages, their rightward deviation is shown to temporarily decrease.^[28]^ When considered together, it is not only gravitational stimulation but also the level of arousal that affects the processing of spatial information. In our study, USN patients in a state of arousal of GCS15 were asked to perform 4 table-top pen-and-pencil tests, each in the supine, sitting, and standing positions in a lighted room. The improvement in their rightward deviation in the supine position was better compared to the sitting position. As we conducted tests in USN patients with a good arousal state in a lighted environment, a reduction in the level of arousal resulted in little effect, even in the supine position. The supine position had the least gravitational stimulation among the 3 positions and resulted in a better improvement of spatial neglect than the sitting position due to the advantageous functioning of the vestibular nervous system. Furthermore, in the supine position, the right hand was used in performing the tests, and muscle contraction of the right upper limb was needed, which may have also contributed advantageously.

In this study, the standing position, in which there is the most gravitational stimulation among the 3 positions and which is considered to cause the most disadvantageous effect on USN, conversely improved USN patients’ rightward deviation better than the sitting position. Thus, factors other than gravitational stimulation may have contributed advantageously to USN in the standing position. In patients in a vegetative or minimally conscious state, the standing position largely affects the level of arousal, consciousness, and behavior.^[29]^ Moreover, in the standing position, due to postural control, the muscular activity in the lower limbs and the trunk becomes larger compared to the sitting position, and afferent signals of the muscle spindles and Golgi tendon organs increase. These signals are thought to reach the reticular activating system,^[30]^ which may contribute to the persistence and improvement of the level of arousal and attention. Therefore, stimulation of muscles and tendons in the standing position causes an advantageous effect on the ascending reticular activating system to elevate the level of arousal and attention compared to the sitting position, where muscle contraction is less needed. This may have led to a better improvement in USN in the standing position than in the sitting position.

In our study, the supine position resulted in a better improvement in rightward deviation compared to the sitting position, and the standing position resulted in a better improvement than the sitting position, whereas there was no difference between the supine and standing positions. In both supine and standing positions, a posture of the extended lumbar spine, hip, and knee joints is maintained, and there is no difference in the positions of the extremities. However, gravitational stimulation, as well as arousal levels, differs. In the supine position, there is little gravitational stimulation, which disadvantageously affects USN, as well as arousing stimulation, which advantageously affects USN, while in the sitting position, much of both are present. The fact that there is a balance between the positive and negative effects of such stimulation on USN in each position may have resulted in similar effects on USN in both positions.

Compared to non-USN stroke patients, USN patients show slower and suppressed recovery patterns of perception/motor disorders and limited activities of daily living.^[31]^ Therefore, improving USN is important in rehabilitation programs for hemiplegic stroke patients. This study demonstrated that the effect of body position should be considered in rehabilitation therapy for stroke where USN is present. The results suggest that performing standing-up and walking training is also effective for USN. In addition, the study suggests that even in the supine position during the acute and convalescent phases of the stroke, tasks causing muscle contraction of the upper limbs are useful for USN.

### 4.1. Study limitations

First, the study did not include a control group of patients without spatial neglect. Second, due to variations in lesion sites, differences in the results caused by lesion sites could not be analyzed. Third, the effect of a somatosensory disorder on the results could not be analyzed. Fourth, the state of consciousness/attention, muscle contraction of the extremities and the trunk, functions of the muscle spindles, and Golgi tendon organs during testing were not assessed.

## 5. Conclusion

The study demonstrated that body position affects USN in stroke patients and that the standing and supine positions improve USN more than the sitting position. Some possible mechanisms are: muscle contractions in the lower limbs and the trunk could have affected results in the standing position, and reduction in gravitational stimulation in the supine position could have played a role. Body position must be considered when conducting rehabilitation therapy for USN.

## Acknowledgments

We would like to thank the medical staff at Wakayama Medical University Hospital for their assistance, especially Mr. Kenji Niji, Mr. Takashi Moriki, Mr. Youhei Terai, Mr. Kenzo Teramura, and Ms. Takako Nougawa. Further, we would like to thank Editage (http://www.editage.com) for English language editing.

## Author contributions

**Conceptualization:** Hitoshi Onaka, Yukihide Nishimura, Fumihiro Tajima, Yukio Mikami.

**Data curation:** Hitoshi Onaka, Ken Kouda, Hidenori Tojo.

**Formal analysis:** Hitoshi Onaka.

**Investigation:** Hitoshi Onaka.

**Methodology:** Hitoshi Onaka, Ken Kouda, Hidenori Tojo, Yukio Mikami.

**Project administration:** Yukihide Nishimura, Yukio Mikami.

**Supervision:** Ken Kouda, Yasunori Umemoto, Toshikazu Kubo, Fumihiro Tajima, Yukio Mikami.

**Validation:** Ken Kouda, Hidenori Tojo.

**Visualization:** Ken Kouda, Hidenori Tojo.

**Writing – original draft:** Hitoshi Onaka, Toshikazu Kubo.

**Writing – review & editing:** Yukihide Nishimura, Ken Kouda, Yasunori Umemoto, Toshikazu Kubo, Fumihiro Tajima, Yukio Mikami.
